# Non-coding genomic regions possessing enhancer and silencer potential are associated with healthy aging and exceptional survival

**DOI:** 10.18632/oncotarget.2877

**Published:** 2015-02-28

**Authors:** Sangkyu Kim, David A. Welsh, Leann Myers, Katie E. Cherry, Jennifer Wyckoff, S. Michal Jazwinski

**Affiliations:** ^1^ Tulane Center for Aging and Department of Medicine, Tulane University Health Sciences Center, New Orleans, LA 70112, USA; ^2^ Department of Medicine, Louisiana State University Health Sciences Center, New Orleans, LA 70112, USA; ^3^ Department of Biostatistics and Bioinformatics, School of Public Health and Tropical Medicine, Tulane University Health Sciences Center, New Orleans, LA 70112, USA; ^4^ Department of Psychology, Louisiana State University, Baton Rouge, LA 70803, USA

**Keywords:** aging, frailty, longevity, linkage, association, non-coding

## Abstract

We have completed a genome-wide linkage scan for healthy aging using data collected from a family study, followed by fine-mapping by association in a separate population, the first such attempt reported. The family cohort consisted of parents of age 90 or above and their children ranging in age from 50 to 80. As a quantitative measure of healthy aging, we used a frailty index, called FI_34_, based on 34 health and function variables. The linkage scan found a single significant linkage peak on chromosome 12. Using an independent cohort of unrelated nonagenarians, we carried out a fine-scale association mapping of the region suggestive of linkage and identified three sites associated with healthy aging. These healthy-aging sites (HASs) are located in intergenic regions at 12q13–14. HAS-1 has been previously associated with multiple diseases, and an enhancer was recently mapped and experimentally validated within the site. HAS-2 is a previously uncharacterized site possessing genomic features suggestive of enhancer activity. HAS-3 contains features associated with Polycomb repression. The HASs also contain variants associated with exceptional longevity, based on a separate analysis. Our results provide insight into functional genomic networks involving non-coding regulatory elements that are involved in healthy aging and longevity.

## INTRODUCTION

Aging can be defined as the occurrence of changes over time that adversely affect the vitality and functions, increasing the mortality rate [1]. The onset of aging varies from individual to individual, and the aging-related changes occur at different rates in different individuals at many levels of biological organization. This complex phenomenon has both genetic and non-genetic underpinnings [2, 3]. The finding that lifespans of various model organisms can be altered, sometimes dramatically, by single gene mutations suggests a role for genes in aging [3, 4]. On the other hand, near doubling of human life expectancy in developed countries during the past two centuries attests to the complexity of the etiology as well as the importance of environmental factors [5].

One quantitative indicator of the genetic basis of a complex trait is heritability, which is a measure of the extent of genetic control of the trait. The heritability of human longevity ranges from 0.15 to 0.35 [6, 7], which also implies that 65–85% of the trait can be controlled by non-genetic factors. However, studies indicate that survival to older ages is under stronger genetic influence. For example, siblings of centenarians are four times more likely to survive to their early nineties compared with siblings of 73-year olds, and siblings of centenarians are at least 8 to17 times more likely to reach age 100 compared with their birth cohort controls [8, 9]. Healthy, long-lived people are likely to carry more beneficial genetic variants, fewer harmful variants or both.

Efforts to find such genetic elements have been made using genetic epidemiological methods [10]. A major hurdle in genome-wide studies of complex diseases is the lack of sufficient statistical power. The lack of power in complex trait studies comes from inadequate setting of a number of important statistical parameters, such as significance level, sample size, and effect size [11, 12]. One approach to alleviate the power demand is to base an experiment on the prior odds generated from the preceding study [11]. A paradigm of this approach is to carry out genome-wide linkage analysis and fine-scaled association mapping of linkage regions [13, 14]. Accordingly, we set up the Healthy Aging Family Study (HAFS) for linkage analysis [15]. Our plan was to dissect linkage-defined regions with association mapping, using our ongoing association studies [16].

For a quantitative measure of heathy aging, we developed the frailty index FI_34_, composed of 34 common health and function variables [15]. FI_34_ increases exponentially with age, indicating declining health and function ability. The rates of increase differ significantly between offspring of long-lived parents (≥ 90 years old) and offspring of short-lived parents (< 76 years old at death), indicating that FI_34_ is associated with parental longevity. The genetic basis of FI_34_ is substantiated by a narrow-sense heritability estimate of 0.39. Using FI_34_, we found elevated levels of resting metabolic rate (RMR) linked to declining health in nonagenarians [17]. This result points to RMR as an important physiological marker of healthy aging among the oldest-old. It also illustrates the use of FI_34_ as a means of identifying additional physiological factors involved in healthy aging.

We have completed genome-wide linkage scanning followed by fine-scale association mapping and found three sites associated with healthy aging at 12q13–14. These HASs are located in intergenic regions. Functional annotation of the HASs indicates that they possess genomic features indicative of enhancer or silencer activity. Our results indicate that healthy aging, and longevity as well, can be controlled by non-coding regulatory elements.

## RESULTS

### A single linkage peak for healthy aging at 12q13–14

**Figure 1:**
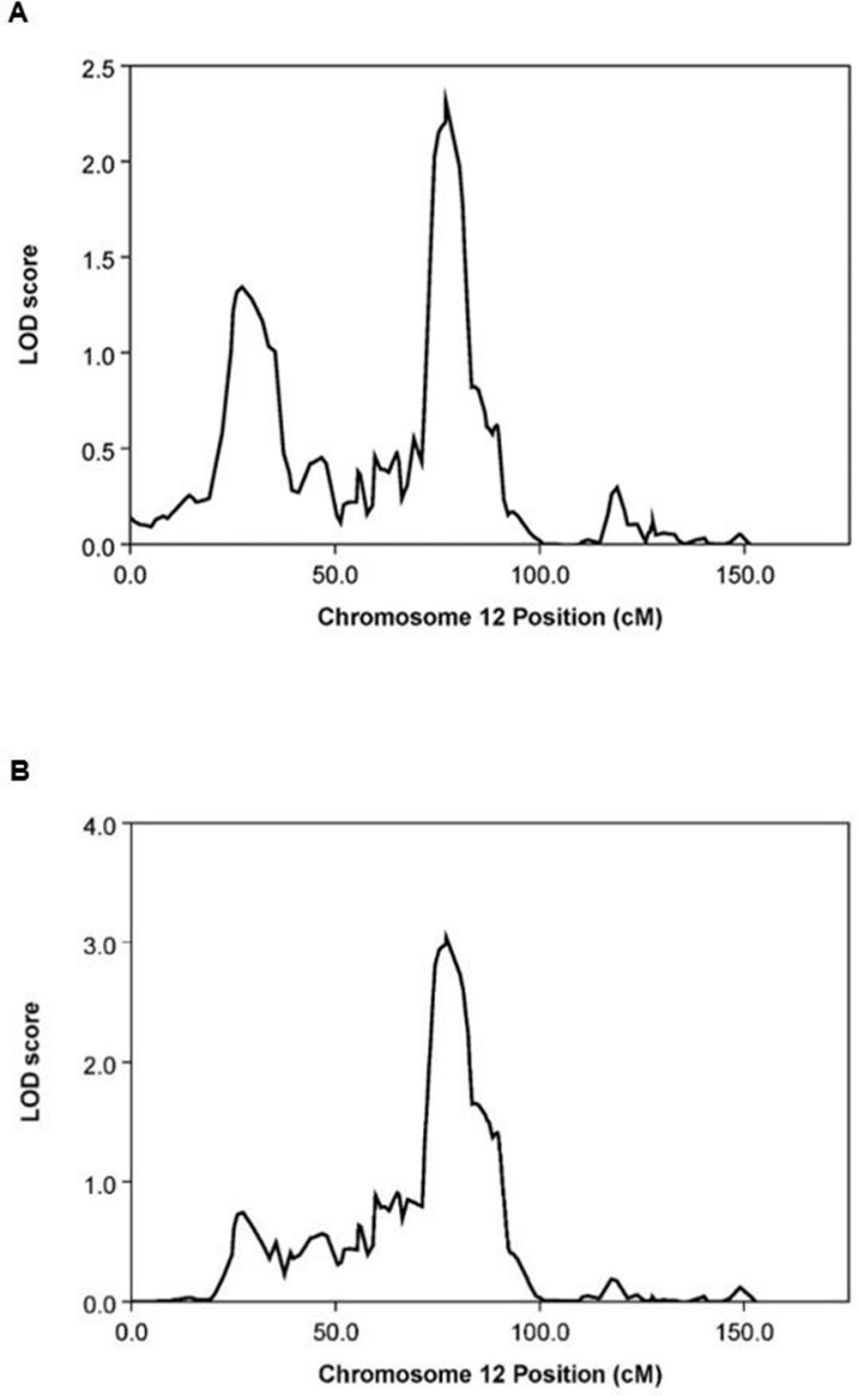
Graphical summary of MERLIN *npl* analysis on chromosome 12 **(A)** From offspring data only (LOD = 2.3, *P* = 6.0 × 10^−4^). **(B)** From offspring data combined with inferred parental phenotype data (LOD = 3.0, *P* = 1.0 × 10^−4^).

When the non-parametric linkage (*npl*) analysis in the MERLIN package was carried out on the data from the HAFS offspring only, the most significant linkage peak was found at 77cM (LOD = 2.3, *P* = 6.0 × 10^−4^) on chromosome 12 (Figure 1A). Because the healthy aging phenotype data were unavailable for HAFS parents, we inferred healthy aging status of each parent from the parent-offspring linear regression, where the slope of the regression line is mathematically equivalent to the narrow-sense heritability of FI_34_ (see METHODS). When the inferred parental data were incorporated in the *npl* analysis, the linkage peak on chromosome 12 became more significant with a higher LOD score (Figure 1B; LOD = 3.0, *P* = 1.0 × 10^−4^). Dense SNP markers can result in inflation of linkage when significant linkage disequilibrium (LD) between adjacent markers exists (see METHODS). However, linkage analysis software assumes marker-marker linkage equilibrium. Therefore, it is important to take into account potential marker-marker LD in linkage analysis. We obtained similar linkage results with different models of LD between SNP markers (Figure S1). Thus, the *npl* analysis delineated a region suggestive of linkage to healthy aging on chromosome 12. The SNPs showing LOD of 3.0 lie in a region of about 1 Mb in size.

### Three healthy-aging associated sites (HAS)

**Figure 2:**
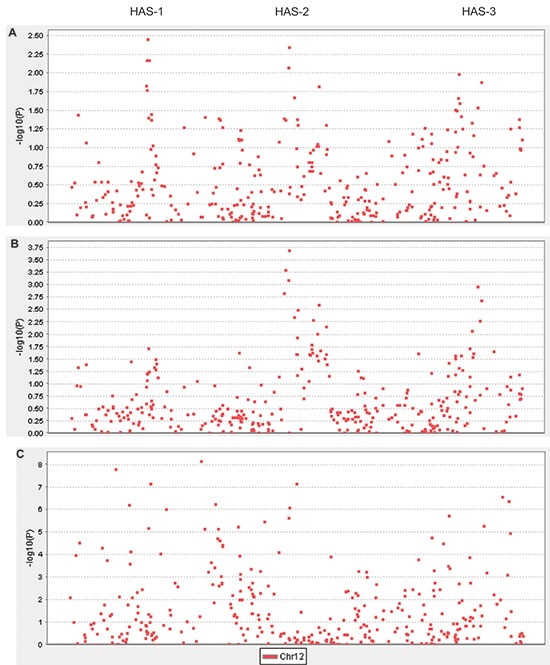
Manhattan plots of association results **(A)** −log_10_ *P* values from linear regressions of FI_34_ scores on additive effects of 330 SNPs, with sex and age differences adjusted, were plotted against SNP positions. Applying a Bonferroni adjustment, the cutoff significance *P* value is 1.52 × 10^−4^ and its −log_10_ counterpart is 3.8. **(B)** The same as in (A) but using logistic regressions of dichotomized FI_34_ values. **(C)** −log_10_ *P* values from χ^2^ tests for differences in allele frequencies between oldest-old cases and young controls were plotted against SNP positions.

For fine-scale mapping, we genotyped 175 LHAS nonagenarians for whom FI_34_ data were available, and applied linear regressions to genotypes of 330 SNPs that are located within the linkage peak (LOD > 2.7). The dependent variable was the FI_34_ score and the independent variable was the genotype of the SNP marker in the additive mode. Age and sex were included as covariates. This way, we tested whether the risk of healthy aging or unhealthy aging among the oldest-old increases additively as the number of copies of the minor allele increases, after adjustment for age and sex. Three groups of SNPs stood out in this analysis (Figure 2A): SNPs in HAS-1 (the lowest *P* = 3.5 × 10^−3^), SNPs in HAS-2 (the lowest *P* = 4.2 × 10^−3^), and SNPs in HAS-3 (the lowest *P* = 1.3 × 10^−2^). Employing the very conservative Bonferroni correction for multiple comparisons to maintain a family-wise error rate of 0.05, no SNP exceeded the adjusted significance level of 1.52 × 10^−4^ (Linear regression in Table 1).

**Table 1:** SNPs associated with healthy aging at 12q13–14

HAS	SNP	Position^†^	Linear regression		Logistic regression	
			Coefficient	*P*	OR	*P*
1	rs10877013^‡^	58165085	−0.023	9.3 × 10^−3^	1.57	7.2 × 10^−2^
	rs10877015	58167788	−0.024	6.6 × 10^−3^	1.59	6.2 × 10^−2^
	rs923829	58174306	−0.026	3.5 × 10^−3^	1.60	5.9 × 10^−2^
	rs6581155^‡^	58178162	−0.024	6.6 × 10^−3^	1.58	7.2 × 10^−2^
2	rs3847663	60542054	0.019	4.2 × 10^−2^	0.36	4.8 × 10^−4^
	rs10784033	60591563	−0.023	8.3 × 10^−3^	2.48	7.8 × 10^−4^
	rs10877403	60605534	0.025	4.5 × 10^−3^	0.36	2.0 × 10^−4^
3	rs7301866	63833069	0.020	2.9 × 10^−2^	0.40	1.1 × 10^−3^
	rs1733676	63873895	−0.012	2.2 × 10^−1^	2.17	5.2 × 10^−3^
	rs7133474	63897318	−0.022	1.3 × 10^−2^	2.32	2.0 × 10^−3^

The Bonferroni-adjusted significance level is 1.52 × 10^−4^ (0.05/330)

† Based on GRCh37s/hg19 assembly

‡ Regression outcomes based on imputed data using IMPUTE2 (v2.3.0)

Because the linkage outcome was based on the binary coding of FI_34_, we dichotomized FI_34_ as the binary status of healthy versus unhealthy aging (see METHODS) and applied logistic regression to the same SNPs with age and sex as covariates. This time, SNPs in HAS-2 and HAS-3 remained conspicuous, whereas HAS-1 became less prominent (Figure 2B). The top SNP in HAS-2 (*P* = 2.0 × 10^−4^) was very close to the significance cutoff set by the Bonferroni adjustment (Logistic regression in Table 1). HAS-1, -2, and -3, delineated by the top four SNPs in each, are ~40 kb, ~340 kb, and ~250 kb long, respectively. SNPs in HAS-1 were previously associated with various diseases [18], whereas the SNPs in HAS-2 and HAS-3 have not been associated with any phenotypes before.

We also examined association of the same set of SNPs with longevity. For this, we genotyped LHAS controls of ages from 39 to 59 and compared allele frequencies between these controls and the nonagenarian cases (Figure 2C). Compared with healthy-aging associated SNPs, more SNPs were associated with longevity from among the 330 chromosome 12q13–14 SNPs genotyped, and many of these associations were highly significant. Also, the longevity-associated SNPs were not limited to the HASs.

### Genomic features of HASs

We examined the genomic features of the healthy-aging associated sites using computational annotation tools. As shown previously [18], a number of protein-coding genes exist in HAS-1, and promoter regions are marked with higher levels of epigenetically modified histones and DNase I sensitive sites compared to non-promoter regions (Figure S2). In addition, the promoters contain transcription factor binding sites that are co-located with chromatin segments indicative of strong enhancer activity. Unlike HAS-1, HAS-2 is largely devoid of any protein-coding genes (Figure S3). However, it contains several features indicative of regulatory elements, such as clusters of H3K4Me1 and H3K27Ac histone marks, DNase I sensitive sites, and transcription factor binding sites. The absence of elevated levels of H3K4Me3, which is usually found near promoters, is consistent with the absence of promoters in HAS-2. All these features coincide with the presence of several strong enhancers across multiple cell lines. In stark contrast with HAS-1 and HAS-2, HAS-3 lacks such active chromatin features (Figure S4). Instead, HAS-3 has multiple Polycomb-repressed blocks in multiple cell lines and a locus encoding a long intergenic non-coding RNA (lincRNA). The full length of this RNA is about 71 kb.

### Functional annotation of SNPs in HASs

**Figure 3:**
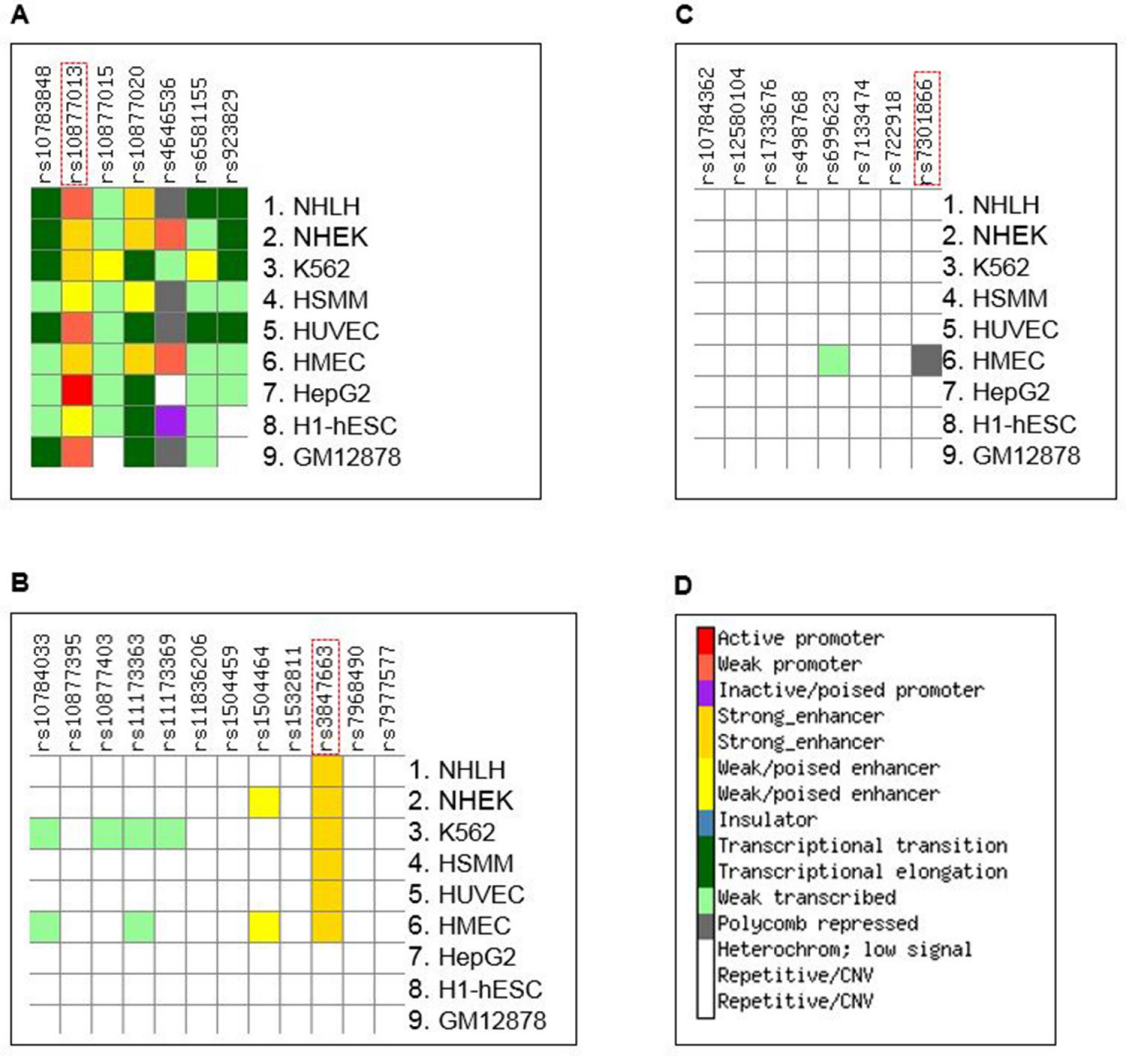
Summary of results of ChroMoS (Chromatin Modified SNPs) annotation for the SNPs in HAS-1 **(A)**, HAS-2 **(B)**, and HAS-3 **(C)** SNPs known or suspected to be functional are enclosed in a dotted red rectangle. **(D)** The genome is functionally segmented into discrete chromatin states through multivariate hidden Markov modeling of ChIP-seq data from multiple cell lines [53]. According to the ‘learned’ chromatin segmentation, ChroMoS graphically assigns individual non-coding SNPs to these chromatin states coded by different colors [19]. Cell lines are NHLF (normal human lung fibroblasts), NHEK (normal human epidermal keratinocytes), K562 (chronic myelogenous leukemia cells), HSMM (human skeletal muscle myoblast cells), HUVEC (human umbilical vein endothelial cells), HMEC (human mammary epithelial cells), HepG2 (human liver carcinoma cells), H1-hESC (human embryonic stem cells), and GM12878 (lymphoblastoid cells).

**Figure 4:**
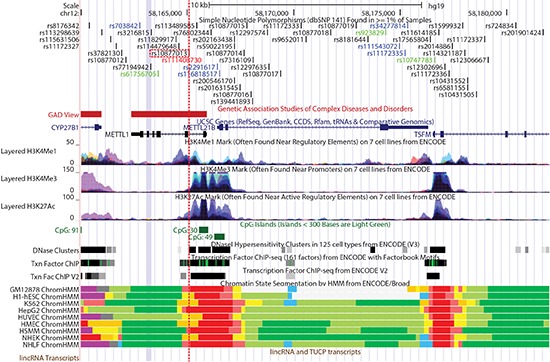
A close-up view of HAS-1 including rs10877013 (red-dotted line) provided by the UCSC Genome Browser [54] SNP IDs in black are in introns, green in coding (synonymous), red in coding (non-synonymous), and blue in untranslated regions. Loci in which variants have been associated with complex diseases or disorders are shown in red blocks. The UCSC Gene track is based on gene prediction data from sources indicated. Coding exons are represented by thick blocks, non-coding or untranslated regions by relatively thin blocks, and introns by thin lines. Gene names and blocks in black represent genes entered in the Protein Data Bank (PDB) and those in blue are transcripts reviewed or validated by either the RefSeq, SwissProt or consensus coding sequence (CCDS) project. Different colors in the histone modification tracks represent results from different cell lines, and peak levels show enrichment levels of the corresponding histone marks as determined by ChIP-seq assays. The numbers following ‘CpG’ represent CpG dinucleotide counts. The DNase Clusters track shows DNase hypersensitive sites with the darkness being proportional to the sensitivity. The ‘Txn Factor ChIP’ track shows transcription factor binding sites from ChIP-seq experiments carried out by the ENCODE project [55]. The DNA binding motifs are from the ENCODE Factorbook repository, which can be viewed as a matrix of all ENCODE transcription factor ChIP-seq datasets, arranged by cell lines [56]. The darkness is proportional to the signal strength, and the green highlights indicate the highest scoring-site motifs. The ‘Txn Fac ChIP V2’ track is similar to the other track, but it employs a different computation method. The ChromHMM tracks, like ChroMoS, show chromatin segments corresponding to different functional states as shown in Figure 3D, according to the computational integration of ChIP-seq data from multiple cell lines using a Hidden Markov Model [57].

**Figure 5:**
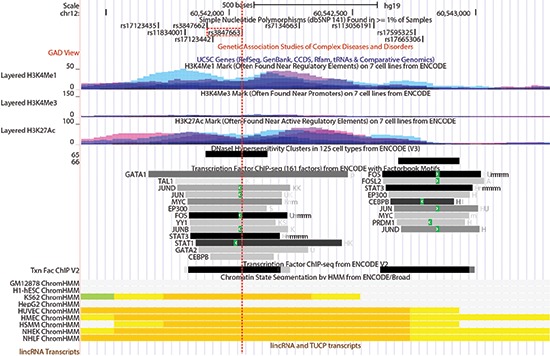
A close-up view of HAS-2 including rs3847663 (red-dotted line) provided by the UCSC Genome Browser Track displays are as described in Figure 4. The names to the left of individual transcription factor binding sites are the HGNC gene names for corresponding transcription factors.

**Figure 6:**
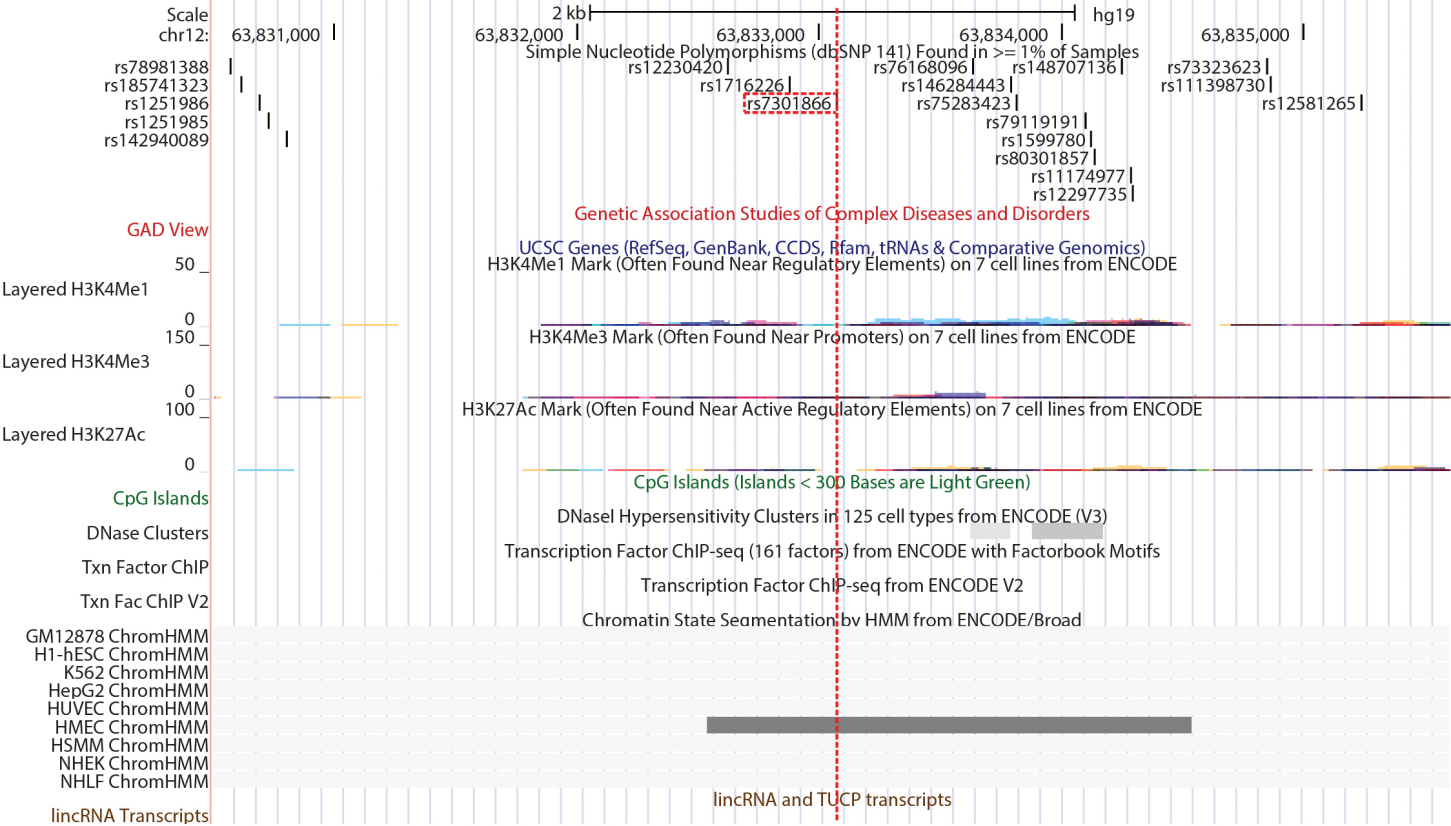
A close-up view of HAS-3 including rs7301866 (red-dotted line) provided by the UCSC Genome Browser The gray-colored block in ChrommHMM tracks represents a Polycomb-repressed site. Other track displays are as described in Figure 4.

Given the non-coding regulatory nature associated with healthy aging, our main objective was to identify the regulatory elements and their target genes. To do so, we searched for functional SNPs that are responsible for the linkage and association outcomes. We used ChroMoS to obtain functional annotations of SNPs present in HASs. ChroMoS assigns putative functions of individual SNPs based on genetic and epigenetic data [19]. Of the SNPs examined for HAS-1, rs10877013 was assigned strong enhancer activity in multiple cell lines (Figure 3A and 3D). Consistent with this annotation, the SNP falls within multiple transcription factor binding sites (Figure 4). For HAS-2, rs3847663 was predicted to overlap a strong enhancer element in various cell lines (Figure 3B and 3D). It falls within stretches of histone marks indicative of active regulatory elements, a DNase I hypersensitive site, and multiple transcription factor binding sites (Figure 5). On the other hand, rs7301866 in HAS-3 was predicted to be in a Polycomb-repressed site in one cell line (Figure 3C and 3D). It is located within a segment subject to Polycomb repression (Figure 6).

## DISCUSSION

According to recent database statistics compiled by LongevityMap, of the total 755 loci studied for their associations with human aging and longevity, 257 were entered as significant [20]. However, most of these genes or variants remain to be validated; only a handful of them, such as *APOE* and *FOXO3A*, have been replicated in separate studies [21]. The number of human aging studies utilizing genetic linkage analysis is much more limited, and most of these linkage results haven't been pursued further. Currently, the only significant linkage peak identified in more than one study is located at 3p24–22 (Table 2).

**Table 2:** Summary of linkage studies on healthy aging and longevity

Phenotype	Location	LOD score (*P*-value)	Reference
Longevity	4q25	3.65 (4.4 × 10^−2^)^a^	[58]
Healthy aging	4q25	1.67 (3.0 × 10^−3^)^b^	[23]
Longevity	3p24.2–22.3	4.02 (3.7 × 10^−2^)^c^	[5]
	9q31.3–34.2	3.89 (5.4 × 10^−2^)^c^	
Successful aging	Chromosome 6,	4.49^d, †^	[24]
	7	3.11^e^	
	14	4.17^e^	
Successful aging	Chromosome 6,	3.24^d, †^	[59]
	10,	4.2^e^	
	16	3.3^d^	
	17,	3.5^e^	
	20	3.3^e^	
Longevity	3p24–22	4.19 (1.0 × 10^−5^)^f^	[60]
Longevity	14q11.2	3.47^g^	[61]
	17q12–22	2.95^g^	
	19p13.3–p13.11	3.76^g^	
	19q13.11–q13.32	3.57^g^	
Healthy aging	12p13–14	3.0 (1.0 × 10^−4^)^h^	This study

a non-parametric (empirical *P* value);

b non-parametric (point-wise *P* value);

c non-parametric, Kong & Cox LOD score – exponential model (empirical *P* value);

d HLOD;

e NPL statistic in MERLIN;

f Z-score (point-wise *P* value);

g non-parametric;

h non-parametric, Kong & Cox LOD score – linear model (point-wise *P* value):

† Different regions

By employing linkage analysis followed by association mapping, we mapped genomic sites on chromosome 12 that are linked to healthy aging, which in consequence uncovered multiple, novel genetic markers of longevity. Our approach deserves several comments. The genome-wide linkage scan is unbiased in that it doesn't need any prior knowledge. Secondly, having found a significant linkage, we were able to focus on the linkage region with a lowered power barrier. Thirdly, our phenotype (expressed as FI_34_) is well defined and characterized, with substantial familial clustering and heritability. Fourth, we leveraged the linkage analysis to establish association of gene variants with the phenotype in a separate cohort.

Typical linkage studies of human aging are carried out on the oldest-old with little consideration of the individual's health and/or function ability (Table 2). Various health deficits begin to accumulate at age 60~70, and older people accumulate deficits at different rates [15, 22]. Thus, elderly individuals of the same age may differ in their healthy-aging status. Because healthy aging is a significant predictor of mortality, the actual life expectancy of these individuals may substantially differ depending on their healthy-aging status. Therefore, we considered it to be more informative and productive to incorporate a validated functional measure into a linkage analysis than to base the analysis solely on chronological age. The number of studies using such a measure of healthy aging is very limited. Reed *et al*. [23] defined the healthy phenotype in their study based on a small number of variables: reaching age of at least 70 and the absence of medical history of several major diseases. On the other hand, similar to our study, Edwards *et al*. [24] incorporated a relatively large number of health variables, including variables for physical and cognitive functioning, to their phenotype of successful aging. Although statistical and genetic properties of their phenotypic measure are not known, we assume that their measure is similar to our well characterized index of healthy aging. The reason underlying this assumption is that as long as the numbers of health variables are statistically sufficient, different frailty indices show similar statistical properties, even if they are based on different types of health variables [25].

Few of the linkage studies have been replicated, which is common in other study designs, especially in population-based association studies [26, 27]. The linkage region at 4q25 was captured early on in two separate studies, but it failed to be corroborated later in another study [28]. Our study is similar to the study by Edwards *et al*. [24] in that both incorporated functional measures of aging. However, the linkage regions identified by the two studies differ. There could be a number of reasons for this, such as variation in study design or in genetic background. One of the characteristics of complex traits is the presence of multiple layers of gene-gene and gene-environment interactions occurring in and between underlying genetic networks [16, 27, 29]. Frequencies of risk variants may vary across different populations, which may lead to variable gene-gene interactions [29]. Moreover, the effect of a gene can be allele- and sex-specific [30]. Consequently, it is not surprising to see different results from different studies involving different populations, where allele frequencies and environments likely differ.

In our study, we examined two separate (though related) phenotypes for the first time, healthy aging and longevity. Somewhat different outcomes were observed for association of SNPs depending on the phenotype. The allelic association tests of longevity involved both young controls and nonagenarian cases, whereas the regression tests of healthy aging involved the oldest-old cases only. Furthermore, the phenotype of healthy aging is not exactly the same as the phenotype of longevity. Although FI_34_ is correlated with mortality/survival, the two statistical association tests need not necessarily converge on the same SNPs. As shown earlier [15], FI_34_ predicts mortality better than does chronological age. Importantly, all the healthy-aging associated sites contained longevity-associated SNPs, and these results were also confirmed by permutation testing with 10,000 replicates.

All the functionally annotated SNPs that we examined in the healthy-aging associated sites (HAS) are non-coding variants. In particular, HAS-2 and -3 are located in intergenic regions barren of any known mRNA-encoding loci. Thus, our foremost task was to assign functionality to the SNPs that could be causative for the healthy-aging association. Many post genome-wide association studies of human pathological traits have found non-coding variants capable of modulating gene expression by affecting transcriptional enhancer or silencer activity [31].

SNP rs10877013 in HAS-1, located within a putative binding site for the CCAAT/enhancer binding protein (C/EBP), affects enhancer activity of DNA fragments containing the SNP in an allele- and orientation-dependent manner [18]. Data from long-range chromatin interaction assays indicate that the targets of this enhancer activity include a number of adjacent genes in HAS-1 [32]. Regarding HAS-2, annotation data indicate that rs3847663 overlaps an enhancer element marked by multiple transcription factor binding sites in the middle of a “gene desert” (Figure 5). Transcriptional enhancers are typically located a few genes away from their target genes or even on different chromosomes. Enhancers may regulate transcription of target genes through long-range interactions, mediated by the formation of chromatin loops [33]. According to the transcriptome data obtained from monocytes [34], several SNPs in HAS-2 are associated with transcription of genes on different chromosomes, with *P* values ranging from 9.96 × 10^−6^ to 1.79 × 10^−7^. Although these eQTL associations are considered not significant in this particular cell type (the study-wise threshold of significance was set at *P* < 5.78 × 10^−12^), these findings suggest that these healthy-aging associated sites may physically interact with other genomic sites to exert their regulatory effects over the course of healthy aging.

HAS-3 is flanked by two genes, *AVPR1A* and *DPY19L2*, whose bivalent promoters are surrounded by Polycomb-group (PcG) protein-repressed sites (Figure S4). It also contains a lincRNA-encoding locus. The human genome encodes more than 3,000 lincRNAs, and PcG complexes are often associated with lincRNAs [35]. At least some of the lincRNAs are known to mediate recruitment of the PcG complexes to target sites for transcriptional silencing [36]. Chromatin modification and compaction mediated by PcG complexes seem to spread in cis from the Polycomb binding sites, affecting nearby genes on the same chromosome [37]. Recently, Pemberton *et al*. [38] carried out RNA-seq and ChIP-seq on human fibroblasts and compiled a list of candidate target genes of Polycomb silencing. According to this data set combined with the ENCODE data, the best candidate genes subject to PcG silencing in HAS-2 are *AVPR1A* or *DPY19L2* or both.

In sum, we have found a novel genomic region that is linked to healthy aging. We have taken this linkage analysis a step further, for the first time, by fine-mapping this genomic region, using a different population sample. Association mapping delineated three sites associated with healthy aging: HAS-1 and -2 seem to possess enhancer activity, whereas HAS-3 has silencer activity. HAS-1 to 3 also contain variants associated with longevity. We envision a mechanism of healthy aging and longevity based on non-coding regulatory elements as upstream regulators of multiple genes. Unraveling this mechanism and identification of the downstream target genes will significantly further our understanding of the etiology of healthy aging and longevity.

## METHODS

### Study subjects

The Healthy Aging Family Study (HAFS) and demographic characteristics of its participants were described elsewhere [15]. The participants are Louisiana residents who were at least 90 years old and their offspring (*N* = 320), recruited in sibships. Ethnicity was self-declared. The Louisiana Healthy Aging Study (LHAS) was also described elsewhere [16]. Its participants are unrelated individuals (*N* = 869), ranging in age from 20 to over 100 years old. Ethnic affiliation was genetically-inferred using Structure analysis (0.8 assignment probability) [16, 39]. Only European-origin participants were included in the analyses to avoid confounding by population admixture. Ages of participants were verified using both documentary evidence (birth certificates, passports, and driver's licenses) and demographic questionnaires. All participants provided informed consent according to protocols approved by the Institutional Review Boards.

### Genotyping

Following genomic DNA extraction and quantification, genotypes of 5,913 biallelic SNPs for 324 subjects were generated, using the Illumina Infinium Linkage 24 set. Genotype data were imported into GenomeStudio Data Analysis Software and analyzed using the Genotyping Module. The starting dataset contained genotype data for 5, 913 SNPs from a total of 320 subjects. The project threshold (GenCall score cutoff) was set at the default value of 0.25, and genotypes of samples with call rates ≥ 0.9 were exported and further analyzed. SNPs not in Hardy-Weinberg proportions (*P* < 0.01 using unrelated subjects) and SNPs with minor allele frequencies below 1% were eliminated using PLINK [40]. Genotyping errors were detected and removed by Mendelian error checking using PEDSTATS [41]. A genetic relationship matrix was constructed using GCTA [42]. Pedigree errors and cryptic relatedness between individuals were further investigated using PREST [43]. The likelihood for detection of pedigree errors increases with the number of genotyped markers, and the use of more than 5,000 genome-wide markers greatly facilitated unequivocal error detection. The PREST output in combination with the genetic relationship matrix and the participant enrollment table were used to correct pedigree errors. The detected errors include the presence of unrelated individuals in families, which is known to commonly occur by sample swabbing and mislabeling, misclassification of relationships within families (*e.g*., claimed full-sib instead of actual half-sib), and an incorrect report of ethnicity. Any genotype errors in the corrected pedigrees were removed by another application of PEDSTATS. The final dataset contains 5, 533 SNPs for 392 subjects, including 98 dummy subjects created for missing parents (only one parent was available in most of the families).

For association mapping, the Illumina GoldenGate assay was performed according to the manufacturer's instructions. SNPs were selected according to the Illumina Assay Design Tool. Following completion of the assay, all the SNPs and samples were analyzed using Illumina GenomeStudio, and the following quality control measures were used: sample call rates ≥ 0.95, SNP call frequency ≥ 0.95, 10% GenCall score = 0.3, Cluster Sep = 0.1 to exclude SNPs with overlapping genotype clusters, AB T mean = 0.2 – 0.8 to exclude SNPs where the heterozygote cluster has shifted toward the homozygotes, AB R mean > 0.8 to exclude SNPs with low intensity data.

### FI_34_ as a quantitative measure of healthy aging

Construction of FI_34_ and its properties were described in detail [15]. The 34 health variables cover various diseases, symptoms, conditions, and functional abilities. They are adrenal disease, anemia, angina, asthma, bathing, body mass index, bronchitis, cataracts, chair stand, congestive heart failure, chronic obstructive pulmonary disease, diabetes, dressing, emphysema, feeding, family history of cancer, Geriatric Depression Scale, heart attack, high blood pressure (at the test), high cholesterol, history of high blood pressure, heart murmur, heart problem, kidney disease, liver disease, Mini-Mental State Exam, osteoporosis, seizure, self-rated health, semi-tandem balance, stroke, thyroid disease, transient ischemic attack, and urinary infection.

### Estimation of parental healthy aging status

We inferred healthy aging status of each parent from offspring data as follows. In selective breeding of animals or plants, response (R) is proportional to selection (S) with the constant of proportionality being the narrow-sense heritability (h^2^) [44, 45]:

$$\text{R}={\text{h}}^{2}\times \text{S}$$ (1)

In other words, the average phenotypic value of offspring (P_off_) is equal to h^2^ times the average phenotypic value of parents (P_par_):

$${\text{P}}_{\text{off}}={\text{h}}^{2}\times {\text{P}}_{\text{par}}$$ (2)

Narrow-sense heritability is the proportion of phenotypic variance (V) accounted for by the additive genetic variance (V_a_):

$${\text{h}}^{\text{2}}={\text{ V}}_{\text{a}}/\text{ V}$$ (3)

With (3), equation (2) becomes

$${\text{P}}_{\text{off}}=\;[{\text{V}}_{\text{a}}/\text{ V}]\times {\text{ P}}_{\text{par}}$$ (4)

V_a_ is the covariance (Cov) between parents and offspring (slope between independent variable x and dependent variable y). Thus, equation (4) becomes

$${\text{P}}_{\text{off}}=\;\left[\text{Cov }/{\text{ V}}_{\text{par}}\right]\;\times {\text{ P}}_{\text{par}}\left({\text{V}}_{\text{par}}=\text{ parental phenotypic variance}\right)$$ (5)

Cov/V_par_ is the regression coefficient. Thus, equation (5) becomes

$${\text{P}}_{\text{off}}=\text{ coefficient }\times {\text{ P}}_{\text{par}}$$ (6)

Thus, h^2^ is equal to regression coefficient (compare (2) and (6)). Rearranging (6) leads to

$${\text{P}}_{\text{par}}={\text{ P}}_{\text{off}}/\text{ coefficient}$$ (7)

Regression of offspring on only one parent underestimates narrow-sense heritability by about 50%. However, our estimate of h^2^ of FI_34_ is based on intraclass correlation involving full sib pairs only [15]. Accordingly, we were able to estimate phenotypic values of parents by dividing offspring phenotypic values by h^2^. For each family, we took the average of offspring FI_34_ scores as the offspring phenotypic value and h^2^ as 0.39, to infer parental FI_34_ at corresponding age. To use the inferred FI_34_ values in MERLIN analysis (see below), parents were divided into two age groups (90–94, 95–104) (Table S1), and parents in each age group were dichotomized using the lower limit of the 95% CI for the mean FI_34_ of each age group as a cutoff, as described below.

### Linkage analysis

To carry out linkage analysis, we used the *npl* module of MERLIN-1.1.2 with –pairs –npl command line options [46]. The –pairs option yields the Whittemore and Halpern NPL *pair* statistic to test for allele sharing among pairs of affected individuals, whereas the –npl option gives the Whittemore and Halpern NPL *all* statistic to test for allele sharing among all affected individuals [46, 47]. In general NPL *all* statistics were better than the *pair* statistics (*i.e*., higher LOD scores and lower *P* values), and the linkage statistics and plots presented in this paper are from the *all* statistics. The *npl* module of MERLIN requires binary traits. The HAFS subjects were divided into four age groups: three offspring age groups (60 to 64, 65 to 69, and 70 to 74) and two parent age groups (90–94, 95–104) (Table S1). The division into age groups accounts for the increase in FI_34_ with age, in the analyses. Offspring in each age group were dichotomized using the lower limit of the 95% CI for the mean FI_34_ of each age group as a cutoff. The reason for using the lower limit instead of the upper limit or the mean was to be more stringent in forming the ‘healthy’ aging group against the ‘unhealthy’ aging group. If an FI_34_ score is lower than the cutoff, the subject is coded ‘2’ (yes) for healthy aging; otherwise, the subject is coded ‘1’ (no). We obtained similar results from using the lower limit of the 90% CI (Figure S5A). With the mean FI_34_ value as the cutoff in each age group, we obtained the same linkage peak, but the LOD score was lower (Figure S5B). The linkage peak at 12q13–14 was not observed in all the *npl* runs when age group 0 (ages < 60) was included (Figure S5C-E).

### Modeling marker-marker LD

The LD modeling in MERLIN is based on haplotype frequency estimation within clusters of markers [48, 49]. We used –rsq 0.16 (or 0.40) along with –grid 2 (or 5) options (Figure S1). The rsq option is to create clusters of adjacent markers for which pairwise r^2^ exceeds 0.16 (or 0.4), and the grid option is to have MERLIN carry out analysis at a 2-cM (or 5-cM) interval along the chromosome. Boyden and Kunkel [50] and Edwards et al. [24] used the r^2^ value of 0.16.

### Association mapping and Manhattan plots

We conducted a case-control association analysis using the LHAS sample. Following quality control measures as described above, we obtained genotype data for 453 SNPs in 312 subjects (136 controls of age from 31 to 59 plus 176 cases of age ≥ 90). The 176 cases were divided into two age groups (90 to 94 and 95 to 103), and the cutoff FI_34_ value for each age group was calculated in the same way as the cutoff values for the *npl* linkage analysis (Table S2). The same binary coding used in the linkage analysis was used: If a FI_34_ score was lower than the cutoff, the subject was coded ‘2’ (yes) for healthy aging; otherwise, the subject was coded ‘1’ (no). All statistical analyses to test SNP associations were performed in PLINK. The additive mode of inheritance for each SNP assumed an increasing effect of its genotype with the increasing dose of the minor allele. The linear regression of raw FI_34_ scores on additive effects of SNPs was performed by issuing –linear command line option along with –covar and –sex options, which were to adjust for age (in a separate covariate file) and sex. Similarly, the logistic regression of dichotomized FI_34_ on additive effects of SNPs was carried out by using –logistic. The association for longevity between cases and controls was carried out by using the standard –assoc option, which generated asymptotic *P* values based on 1-df χ7;^2^ statistics. We also performed Fisher's Exact test using –fisher option and permutation by –perm option. The PLINK outputs were directly fed into Haploview (v4.2) [51] to construct Manhattan plots.

### Genotype imputation

IMPUTE2 (v2.3.0) [52] was used to impute genotypes for SNPs that were not included in the genotyping assay. Before using IMPUTE2, PLINK input files were prepared for SNPs that were located adjacent to the un-typed SNPs whose genotypes were to be imputed. The PLINK files were converted to the IMPUTE2 file format using GTOOL (v0.7.5) (http://www.well.ox.ac.uk). Imputation was carried out as directed by the user guide, using reference files downloaded from https://mathgen.stats.ox.ac.uk/impute/impute_v2.html#reference. The output files were converted to PLINK files for association tests.

## SUPPLEMENTARY TABLES AND FIGURES


