# Supplementary material for: Non-coding genomic regions possessing enhancer and silencer potential are associated with healthy aging and exceptional survival
Source: Oncotarget. 2015 Feb 28;6(6):3600–12. doi: 10.18632/oncotarget.2877 (PMC4414140; doi:10.18632/oncotarget.2877)
Supplement: Supplementary file 1 [file oncotarget-06-3600-s001.pdf]

SUPPLEMENTARY TABLES AND FIGURES

Supplementary Table S1: Cutoff values of FI_{34} used to dichotomize samples in each age group of HAFS subjects. Cutoff values are from mean(FI_{34}) – $t_{0.05}$ (or $t_{0.1}$) x sd(FI_{34}) / $\sqrt{n}$ where $t_{0.05}$ is the *t*-value corresponding to the probability of 0.05 for n-1 degrees of freedom. '1' denotes unhealthy aging (equal to or above the cutoff) and '2' healthy aging (below the cutoff)

Age group	0	1	2	3	4	5
Age range	43–59	60–64	65–69	70–84	90–94	95–104
n	46	54	62	36	64	32
Mean(FI_{34})	0.1028	0.1278	0.1469	0.1553	0.2413	0.2692
'1': '2'	20: 26	22: 32	28: 34	16: 20	32: 31	17: 13
sd(FI_{34})	0.0499	0.0773	0.0769	0.0514	0.1336	0.1418
Cutoff–0.05	0.0904	0.1102	0.1306	0.1408	0.2130	0.2267
'1': '2'	27: 19	28: 26	35: 27	22: 14	34: 29	19: 11
Cutoff–0.1	0.0932	0.1141	0.1342	0.1441	0.2193	0.2364
'1': '2'	25: 21	25: 29	33: 29	19: 17	33: 30	19: 11

SupplementaryTable S2: Dichotomization of FI_{34} of LHAS cases for association mapping

Age group	1	2
Age range	90–94	95–103
n	150	26
mean(FI_{34})	0.2216	0.2715
sd(FI_{34})	0.0786	0.1018
Cutoff–0.05	0.2089	0.2302
'1': '2'	74: 76	21: 5

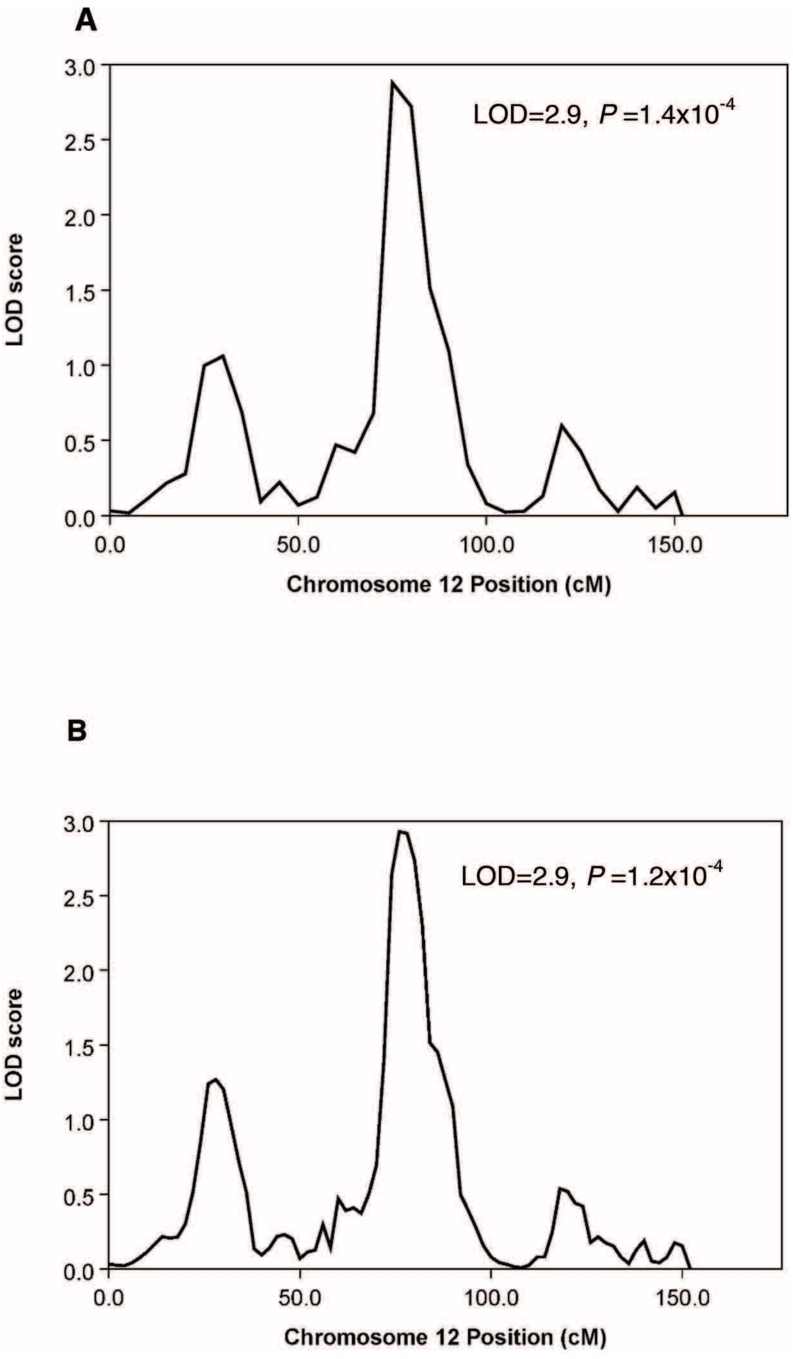

Supplementary Figure S1: Graphical summary of MERLIN *npl* analysis on chromosome 12. **(A)** Analysis of binary healthy aging data (cutoff = 0.05) with –npl –rsq 0.16 –grid 5 command line options. **(B)** Analysis of binary healthy aging data (cutoff = 0.05) with –npl –rsq 0.40 –grid 2 options.

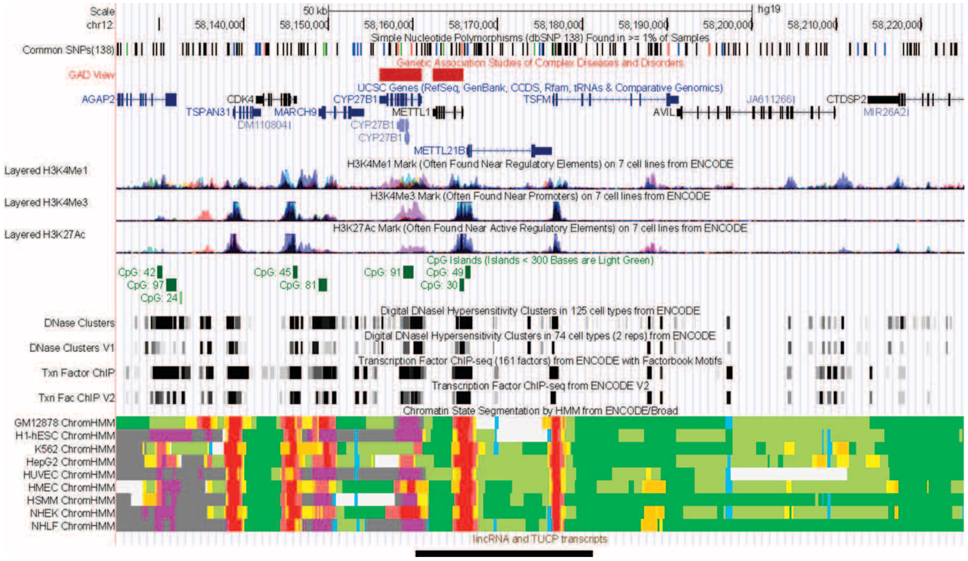

Supplementary Figure S2: A zoomed-out view of HAS-1 provided by the UCSC Genome Browser. In ChrommHMM tracks, orange-colored blocks represent sites for strong enhancers, yellow for weak/poised enhancers, blue for insulators, green for weakly transcribed sites, and red for active promoters. For other track descriptions, refer to the Figure 4 legend. The portion of the site zoomed-in in Figure 4 is shown with a black horizontal bar at the bottom of the figure.

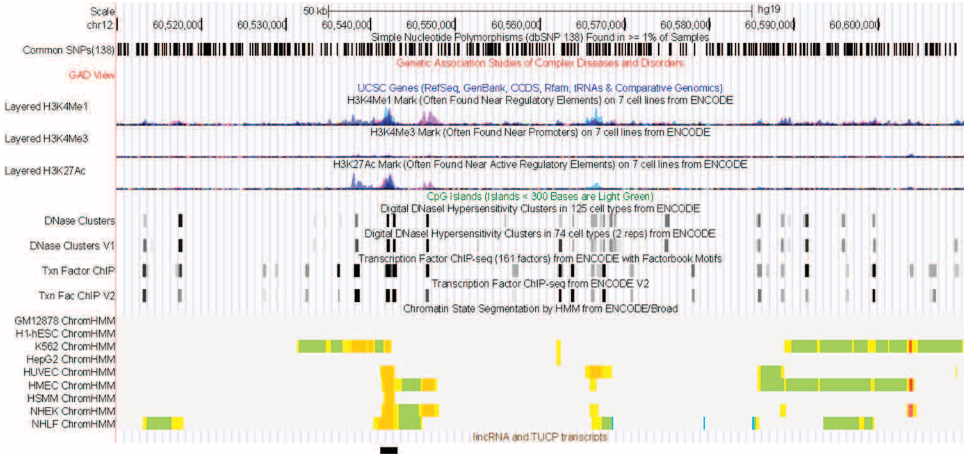

Supplementary Figure S3: A zoomed-out view of HAS-2 provided by the UCSC Genome Browser. In ChrommHMM tracks, orange-colored blocks represent sites for strong enhancers, yellow for weak/poised enhancers, blue for insulators, green for weakly transcribed sites, and red for potential active promoters. For other track descriptions, refer to the Figure 4 legend. The portion of the site zoomed-in in Figure 5 is shown with a black horizontal bar at the bottom of the figure.

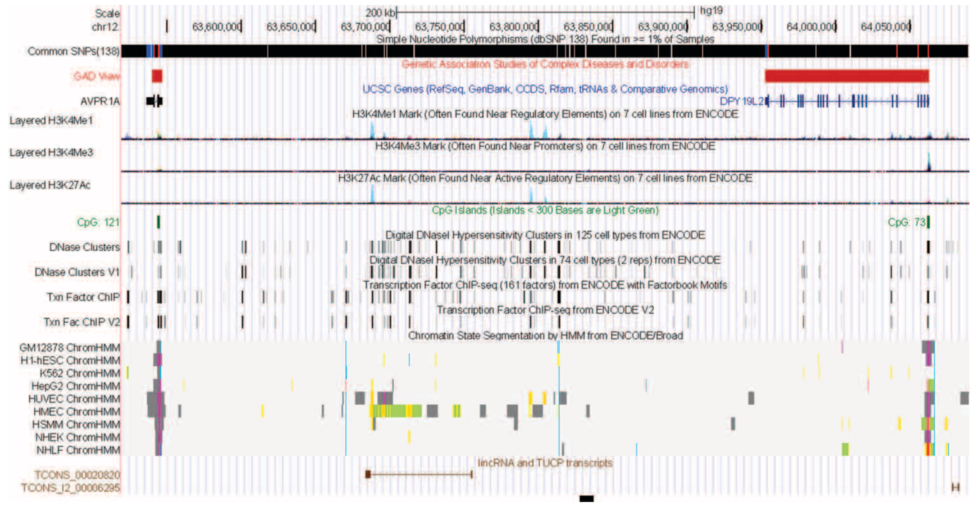

Supplementary Figure S4: A zoomed-out view of HAS-3 provided by the UCSC Genome Browser. In ChrommHMM tracks, the gray-colored blocks represent sites for Polycomb-repressed sites. The purple blocks shown in the promoter regions of *AVPR1A* and *DPY19L2* represent inactive/poised promoters Track descriptions are as described in Figure 4 legend. The track of lincRNA (large intergenic non coding RNA) and TUCP (transcripts of uncertain coding potential) transcripts are from the Human Body Map catalog (www.broadinstitute.org) incorporated in the genome browser. The portion of the site zoomed-in in Figure 6 is shown with a black horizontal bar at the bottom of the figure.

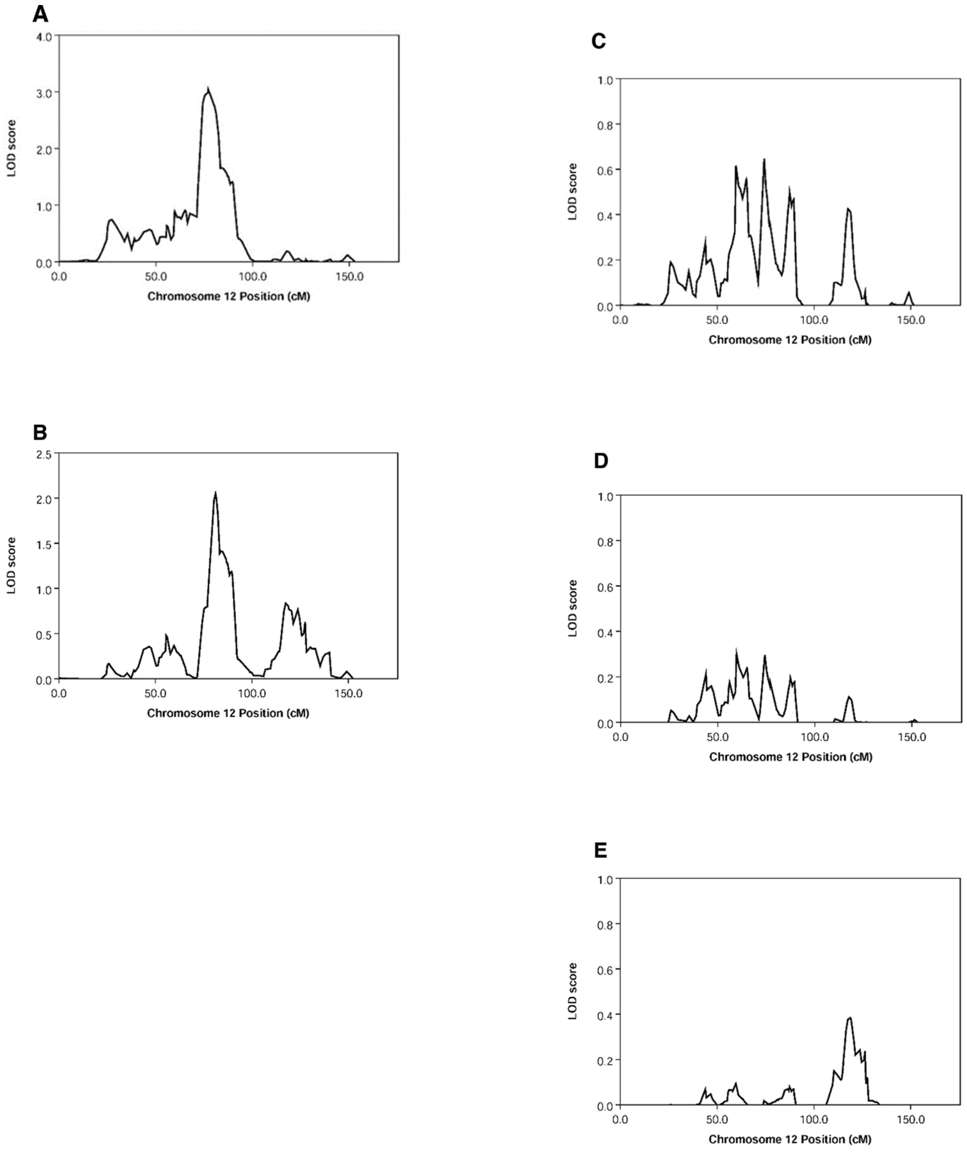

Supplementary Figure S5: Graphical summary of MERLIN *npl* analysis on chromosome 12. **(A)** Analysis of genotype data with binary FI_{34} dichotomized using cutoff-0.1 in each of age groups 1 to 5 (Table S1). **(B)** Analysis of genotype data with binary FI_{34} dichotomized using cutoff-0.5 (mean) in each of age groups 1 to 5. **(C)** Analysis of genotype data with binary FI_{34} dichotomized using the cutoff-0.05 value in each of age groups 0 to 5 (Table S1). **(D)** Analysis of genotype data with binary FI_{34} dichotomized using the cutoff-0.1 value in each of age groups 0 to 5. **(E)** Analysis of genotype data with binary FI_{34} dichotomized using the cutoff-0.5 value in each of age groups 0 to 5.
